# Forum Theatre for Reconciliation: a drama-based approach to conflict transformation applied to socio-environmental struggles in Bolivia

**DOI:** 10.1080/21647259.2024.2351709

**Published:** 2024-05-29

**Authors:** Angelo Miramonti, Lorenza B. Fontana, Caleb Johnston

**Affiliations:** a Bellas Artes, Institución Universitaria del Valle, Cali, Colombia; b Inter-university Department of Regional & Urban Studies and Planning, University of Turin, Turin, Italy; c Collegio Carlo Alberto, Turin, Italy; d Politics, University of Glasgow, Glasgow, UK; e Geography, Newcastle University, Newcastle upon Tyne, UK

**Keywords:** Forum Theatre, conflict transformation, reconciliation, Bolivia, wildfire, climate change

## Abstract

This paper examines how participatory theatre methods can be used to foster reconciliation and conduct research in communities affected by socio-environmental conflicts. We design and pilot a distinctive drama-based approach to conflict transformation that we call Forum Theatre for Reconciliation (FTR). The method furthers embodied intersubjective understanding as well as a critical analysis of structural causes of conflict. We discuss this approach applied to low-intensity communal conflicts linked to extreme wildfires in Bolivia, where fires exacerbate tensions between different communities and result in (sometimes) violent confrontation and polarising discourse. We identify three key innovations of this peacebuilding approach: rehumanising the Self and Other, co-creating complex and inclusive narratives of conflict, and devising collective response across different perspectives and experiences.

## Introduction

Drama-based action research has been increasingly applied across the social sciences with the aim of combining knowledge generation with positive social transformation.^1^^1^For example, see Helen Cahill, ‘Research Acts: Using the Drama Workshop as a Site for Conducting Participatory Action Research’, *NJ* 30, no. 2 (2006): 61–72; Gina Grandi, ‘Theatre as Method: Performance Creation through Action Research’, *Action Research* 20, no. 3 (2022): 245–60; Caleb Johnston and Geraldine Pratt, *Migration in Performance: Crossing the Colonial Present* (New York: Routledge, 2019); Patricia Leavy, *Method Meets Art: Arts-based Research Practice* (New York: The Guilford Press, 2009); Patricia Leavy, *Fiction as Research Practice* (Walnut Creek: Left Coast Press, 2013); Johnny Saldaña, ‘Ethnodrama and Ethnotheatre: Research as Performance’, in *The SAGE Handbook of Qualitative Research*, ed. N. Denzin and Y.S. Lincoln (London: SAGE, 2018); and Ditte Tofteng and Mia Husted, ‘Theatre and Action Research: How Drama can Empower Action Research Processes in the Field of Unemployment’, *Action Research* 9, no. 1 (2011): 27–41. Forum Theatre (FT) has been one of the most popular approaches to addressing social inequalities and injustices through the performative arts.^2^^2^Augusto Boal, *Games for Actors and non-Actors* (New York: Routledge, 2002). FT is a theatrical form systematised by the Brazilian director Augusto Boal as part of an umbrella of techniques called Theatre of the Oppressed.^3^^3^Augusto Boal, *Theatre of the Oppressed* (London: Pluto Press, 1979). It engages vulnerable communities in the search for strategies to break the oppressions they experience. FT has been extensively used to tackle violence and discrimination,^4^^4^Kelly Howe, Julian Boal, and José Soeiro, *The Routledge Companion to Theatre of the Oppressed* (London: Routledge, 2021). conduct social research^5^^5^Luisa Enria, ‘Co-producing Knowledge through Participatory Theatre: Reflections on Ethnography, Empathy and Power’, *Qualitative Research* 16, no. 3 (2016): 319–29; Nick Hammond, ‘Introducing Forum Theatre to Elicit and Advocate Children’s Views’, *Educational Psychology in Practice* 29, no. 1 (2012): 1–18; and Geraldine Pratt and Caleb Johnston, ‘Putting play to work’, in *Politics and Practice in Economic Geography*, eds A. Tickell et al. (London: SAGE, 2007), 71–81. and for conflict transformation.^6^^6^Jeff Aguiar, ‘Applied Theatre in Peacebuilding and Development’, *Journal of Peacebuilding & Development* 15, no. 1 (2020): 45–60; Ananda Breed, et al., ‘Mobile Arts for Peace (MAP): Creating Art-based Communication Structures Between Young People and Policymakers from Local to National Levels’, *Research in Drama Education: The Journal of Applied Theatre and Performance* 27, no. 3 (2022): 304–21; Nilanjana Premaratna, ‘Theatre for Peacebuilding: Transforming Narratives of Structural Violence’, *Peacebuilding* 8, no. 1 (2020): 16–31; and Lena Slachmuijlder and D. Tshibanda, *Participatory theatre for conflict transformation: Training manual. Search for Common Ground*, (2019), https://cnxus.org/wp-content/uploads/2022/04/Participatory-Theatre-Manual-EN.pdf

Our research contributes to debates concerning ‘embodied reconciliation’^7^^7^Roddy Brett, et al., ‘Embodied Reconciliation: A new Research Agenda’, *Peacebuilding* 10, no. 4 (2022): 1–19. by introducing a new approach to participatory theatre for peacebuilding, which we call Forum Theatre for Reconciliation (FTR). Grounded in the classic model of FT, our approach enriches an established FT repertoire with methodological innovations designed to foster reconciliation in conflict settings, specifically addressing the challenges of working with groups who experience oppression in radically different ways and from conflicting perspectives. The contribution of this paper is twofold: first, we systematise our FTR approach and discuss its potential as a peacebuilding tool. Secondly, we illustrate how these techniques can be applied to conduct action research on socio-environmental conflict. The latter is particularly valuable considering the growing concerns about the effects of climate change on social cohesion and the shift towards more fluid, complex and domestic-driven conflicts in Latin America and beyond.^8^^8^William Avis, *Current Trends in Violent Conflict* (*K4D Helpdesk Report*, 2019), https://assets.publishing.service.gov.uk/media/5cf669ace5274a07692466db/565_Trends_in_Violent_Conflict.pdf; and Fernando Calderón et al., *Understanding Social Conflict in Latin America* (UNDP, 2013), https://www.undp.org/sites/g/files/zskgke326/files/migration/latinamerica/Understanding-Social-Conflict-in-Latin-America-2013-ENG.pdf The article reflects on *Playing with Wildfires* (2020–22), an action-research project which used FTR to work with communities experiencing increasing social tensions which stem from extreme wildfires in rural Bolivia (see https://playingwithwildfire.org). The aims of this project were to understand how wildfire crises affect social relationships and communal conflict and to foster dialogue and peacebuilding among local communities.^9^^9^Alastair Cole et al., ‘On Burning Ground: Theatre of the Oppressed and Ecological Crisis in Bolivia’, *Cultural Geographies* 30, no. 4 (2023): 639–48; and Lorenza Fontana et al., ‘Women in Wildfire Crises: Exploring Lived Experiences of Conflict through Forum Theatre’, *Studies in Social Justice* 17, no. 2 (2023): 269–79.

In recent years, Bolivian eastern lowlands, particularly the Chiquitania region, have been among the worst affected by wildfires in Latin America and worldwide. According to the NASA Earth Observatory,^10^^10^NASA, *Uptick in Amazon Fire Activity in 2019* (NASA Earth Observatory, 2019), https://earthobservatory.nasa.gov/images/145498/uptick-in-amazon-fire-activity-in-2019. 2019 saw a noticeable increase in large, intense, and persistent fires throughout the Amazon basin. In Chiquitania, the total burned area was over 5 million hectares; this is five times greater than the previous year and the largest area burned over the past 20 years.^11^^11^Fundación Tierra, *Balance de los incendios forestales 2019 y su relación con la tenencia de la tierra* (La Paz: Fundación Tierra 2019), https://www.ftierra.org/index.php/publicacion/documentos-de-trabajo/attachment/195/52. The exceptional devastation of recent fires, especially in protected areas and indigenous territories, has strained relations between different social groups, including indigenous peoples, migrant peasants, ranchers, Mennonites, and agribusinesses. Whether and how tensions between these groups are managed effectively will likely affect their long-term coexistence and stability, in a context characterised by high level of inter-ethnic conflict.^12^^12^Lorenza Fontana, *Recognition Politics: Indigenous Rights and Ethnic Conflict in the Andes* (Cambridge: Cambridge University Press, 2023); Lorenza Fontana, ‘The «Proceso de Cambio» and the Seventh Year Crisis: Towards a Reconfiguration of the Relationship between State and Social Movements in Bolivia’, *Bolivian Studies Journal* 41, no. 3 (2012): 190–212.

With the hope of facilitating peaceful dialogue among fire-affected communities, while gathering insight into the lived experiences of conflict, we designed an intervention using a FTR approach. This method builds on the insights gained by applying Theatre of the Oppressed and FT to work with people affected by armed conflicts in Colombia. It is the first time (to our knowledge) that this approach is systematized and applied to socio-environmental conflicts. In the paper, we illustrate how this contextualisation of FT in highly polarised settings can support de-enemification, the co-creation of inclusive narratives and the identification of collective responses to address the root causes of conflict. More generally, we are interested in how participatory theatre can be used for conflict transformation in polarised and stigmatised communities.

We describe our work with a mixed group including members of communities in conflict. This process faced challenges and complexities linked to the way in which it is common for different oppressed groups experiencing wildfires in Bolivia to hold each other responsible for fire-related damages. In other words, there was no common and shared ‘oppressor’ that could be identified among vulnerable groups, as is often assumed in FT interventions. We discuss three methodological innovations of FTR to address these challenges, highlighting both the differences between FTR and standard FT as well as the contributions of FTR to the Pedagogy and Theatre of the Oppressed theoretical framework. These include: a) the re-humanisation of the self and the other through the embodiment of the characters belonging to the opposing group; b) the co-creation of a complexified and inclusive narrative around the immediate and structural causes of the conflict; and c) the identification of collective responses across socio-economic divisio. We illustrate each innovation with empirical examples from our Bolivia’s case study.

## Forum Theatre: an ongoing call for contextualisation

In classic FT, a group of performers present a 10- to 15-minute play staging an unresolved oppressive situation familiar to the audience. After the play, a facilitator (called the joker) invites the audience to share opinions about what the protagonist(s) on stage could do to break their oppression. The joker gathers ideas before inviting audience members (reframed as *spect-actors*) to come on stage, replace the protagonist and rehearse the strategies they believe could break character’s oppression. After each rehearsal, the joker often invites the audience to critically assess the proposed strategy by asking two questions: ‘did this strategy manage to break the character’s oppression?’ and ‘does this strategy work only on stage or could it work also in real life?’ Boal conceived FT as a ‘rehearsal of the revolution’ for vulnerable people to understand and confront the structural and behavioural conditions of oppression. Boal highlighted the need to adapt and contextualise FT in response to varied socio-cultural and history contexts, while preserving the ‘irreducible core’ of Theatre of the Oppressed as a method to fight oppression in all its forms.^13^^13^Adrian Jackson, *What is the essence of Theatre of the Oppressed?* (Cardboard Citizens, 2020), https://www.youtube.com/watch?v=46Om7mAz6Kk Since its invention in 1973, FT has been contextualised in a wide range of settings, and both qualitative and quantitative assessments have validated its effectiveness in triggering social, behavioural and political change.^14^^14^See Nick Hammond, ‘Introducing Forum Theatre to Elicit and Advocate Children’s Views’, *Educational Psychology in Practice* 29, no. 1 (2012): 1–18; Karla Hoff, Jalan Jalan, and Santra Sattwik, *Participatory Theater Empowers Women. Evidence from India* (World Bank: Policy Research Working Paper 9680, 2021), http://hdl.handle.net/10986/35642; and Pratt and Johnston, ‘Putting Play to Work’. In particular, FT has demonstrated to be an effective participatory tool for environmental research and conflict transformation. Focusing on FT as a peacebuilding tool to transform socio-environmental conflicts,^15^^15^Neil Adger, ‘Social and Ecological Resilience: Are they Related?’, *Progress in Human Geography* 24, no. 3 (2000): 347–64. Adger^16^^16^Ibid. argues that intercommunal conflict plays a key role in hindering effective response to the climate crisis and calls for establishing a strong link between protracted low-intensity conflict, community resilience and climate change response. Sullivan and Lloyd^17^^17^J. Sullivan and R. Lloyd, ‘The Forum Theatre of Augusto Boal: A Dramatic Model for Dialogue and Community-Based Environmental Science’, *Local Environment* 11, no. 6 (2006): 627–46. share similar sentiments, developing a version of FT called Community Environmental Forum Theatre, integrating FT and Freire’s democratising dialogue^18^^18^Paulo Freire, *Pedagogy of the Oppressed* (The Continuum International Publishing Group, 1970). to conduct environmental research and promote community engagement in Texas. More recently, Brown et al.^19^^19^Katrina Brown et al., ‘The Drama of Resilience: Learning, Doing, and Sharing for Sustainability’, *Ecology and Society* 22, no. 2 (2017): n.p. illustrate how theatre can be instrumental to engaging communities in participatory research and policy design to assess the risks of extreme weather events and build resilience of coastal communities in Kenya and the United Kingdom. Even closer to our endeavour, Inyang and Ebewo^20^^20^Ofonime Inyang and Patrick Ebewo, ‘(Dis) Playing Fear, (dis) Placing Fear: A Theatre-based Strategy for Environment-Related Conflict Management in Rural Nigeria’, in *Applied Drama/Theatre as Social Intervention in Conflict and Post-Conflict Contexts*, eds. Hazel Barnes and Marie-Heleen Coetzee (Newcastle upon Tyne: Cambridge Scholars Publishing, 2014), 48–1. describe the deployment a Theatre for Development framework to facilitate dialogue between youth and elder members of a rural community in Nigeria clashing on the use of forestry resources.

While participatory theatre and in particular FT has been increasingly used to tackle environmental issues, it is less common for the method to consider horizontal intercommunal conflict. One reason could be that the practical tools and epistemological premises offered by classic Theatre of the Oppressed fall short when applied to conflict transformation in highly polarised communities. By labelling some characters and groups as ‘the oppressed’ and others as ‘the oppressor’, the FT dramatic structure can implicitly convey a binary, oversimplifying and de-responsabilising interpretation of the conflict. FT dramaturgy can itself become a field of confrontation in which each conflicting group self-identifies as the ‘victim’ and blames the other for being the ‘aggressor’.^21^^21^David Diamond, *Theatre for Living. The Art and Science of Community-Based Dialogue* (Trafford Publishing, 2007); Adrian Jackson, ‘Interview with David Diamond’ (Cardboard Citizens, 2020), https://www.youtube.com/watch?v=Ibs6-i1yysA. This dichotomising oppressor-oppressed terminology can be exacerbated in situations when social and political leaders manipulate media and collective narratives to increase social tensions and polarisation. Moreover, environmental issues generally linked to the use and management of natural resources could add a level of complexity to the oppressor-oppressed dialectic, whereby root causes can follow timeframes and have impacts which are harder to pin down and identify by local dwellers. This can be linked to climate change, but also to the consequences of economic and political strategies heavily reliant on resource exploitation.^22^^22^Maristella Svampa, ‘Commodities Consensus: Neoextractivism and Enclosure of the Commons in Latin America’, *South Atlantic Quarterly* 114, no. 1 (2015): 65–82; and Eduardo Gudynas, *Extractivisms: Politics, Economy and Ecology* (Black Point: Fernwood Publishing, 2020). In this context, FT could run the risk of fostering the cognitive and emotional process of reciprocal ‘enemification’ between parties in conflict,^23^^23^Robert W. Rieber, *The Psychology of War and Peace: The Image of the Enemy* (New York: Plenum Press, 1991). overlooking their potential interconnectedness and shared responsibilities, as well as broader bewildering causes. This risk is particularly high in contexts where a binary understanding of conflict is deeply entrenched in the collective narratives of each group and a sense of belonging to a collective self is interiorised by the parties as an essentialized identity opposed to the enemy’s identity.

To address these risks, some practitioners have experimented with various contextualisation of FT for conflict transformation and peacebuilding. Chen Alon^24^^24^Chen Alon, ‘Non-Violent Struggle as Reconciliation. Combatants for Peace: Palestinian and Israeli Polarized Theatre of the Oppressed’, in ‘*Come Closer’: Critical Perspectives on Theatre of the Oppressed*, eds. Toby Emert and Ellie Friedland (Peter Lang, 2011), 161–72. developed an adaptation of FT called ‘Polarized Theatre of the Oppressed’. Working with a mixed group of Israeli and Palestinian former combatants, he staged performances at Israeli army checkpoints in the Occupied Palestinian Territories, engaging Palestinian communities, Israeli settlers and soldiers in ‘improbable dialogues’ between enemies.^25^^25^John Paul Lederach, *The Moral Imagination: The Art and Soul of Building Peace* (Oxford: Oxford University Press, 2005). Alon’s reworking of the method does not only search for situational solutions but also aims to create dialogue between communities who live together, and find themselves in conflict.^26^^26^Adrian Jackson, Interview with Chen Alon (Cardboard Citizens, 2020), https://www.youtube.com/watch?v=7HUKCzpoRsI&t=1591s. The ultimate goal of Alon’s approach is to build a ‘third narrative’, co-created through the encounter and reciprocal re-humanisation of the two groups. Another attempt to overcome the risk of polarisation in FT is the ‘Reclaiming our spirits’ project conducted by David Diamond in 1996, in eleven First Nations communities of Canada. Diamond staged the stories residential schoolsurvivors for children in First Nations communities,^27^^27^See https://theatreforliving.com/past_work/reclaiming_our_spirits.htm. depicting lifelong psychosocial consequences of settler colonial violence. The project demonstrated that when survivors became parents, they tended to unconsciously reproduce abuses they suffered as children. However, these survivors refused to be depicted as monsters because they were abusing their children and asked to make explicit (on stage) that their violence was a consequence of their previous victimisation. They also expressed their need to break the silence on residential schools in Canada while seeking dialogue and reconciliation with the descendants of colonisers.^28^^28^Jackson, Interview with David Diamond. In this context, Diamond decided to abolish the oppressor-oppressed terminology and address oppression not as the transitive result of the action of one character or group over the other, but as a system of relations in which each party holds different responsibilities in reproducing the *status quo*.^29^^29^Diamond, *Theatre for Living.*

Building on these experiences, our research aims to contribute to the ongoing effort of contextualising FT to peacebuilding and conflict transformation. We propose a distinctive drama-based approach we call Forum Theatre for Reconciliation and present a pilot experience, where we applied this method to foster peacebuilding in communities affected by socio-environmental conflict in Bolivia.

## Wildfire conflicts in Bolivia: case and method

Fire crises are complex emergencies that gained visibility and became more severe under the effects of global warming, demographic changes, and resource-intensive development strategies.^30^^30^Stefan Doer rand Cristina Santín, ‘Global Trends in Wildfire and its Impacts: Perceptions versus Realities in a Changing World’, *Philosophical Transactions of the Royal Society B: Biological Sciences* 371, no. 1696 (2016): 20150345. In 2019, exceptional wildfires across the Amazon basin had devastating consequences on people and the environment. While Brazil has dominated international media, other neighbouring countries have also suffered similar emergencies. In Bolivia, fires have had a greater proportional impact, with a total burned area similar to Brazil in a country that is eight times smaller.^31^^31^NASA, ‘Uptick in Amazon Fire Activity in 2019’. Most of the destruction in Bolivia was concentrated in Chiquitania, a region that hosts one of the largest and best-preserved dry forests in South America.^32^^32^Alfredo Romero-Muñoz et al., ‘Fires Scorching Bolivia’s Chiquitano Forest’, *Science* 366, no. 6469 (2019): 1082.

Chiquitania represents a multicultural landscape with four main lowland indigenous groups living in the area. Since the early 2000s, the region has received considerable migration of highland indigenous peasants moving eastwards in search of land, with many of the local villages tripling their populations.^33^^33^International Organization for Migration, *Migración Interna en Bolivia* (International Organization for Migration, 2018), https://repository.iom.int/handle/20.500.11788/2111. Chiquitania has also seen exponential growth in livestock production over the past decade, driven by growing export demand. This has been mostly supplied by small and medium ranchers engaged in extensive farming and grazing.^34^^34^FAN, ‘Minería y Ganadería en la Chiquitanía’, *Infofan* (October-December, 2014). Major contributors to this industry are the region’s 46 Mennonite communities, descendent of early-20th-century European migrants. Legal and illegal logging and mining have also expanded in recent years.^35^^35^Alfredo Torrico et al., *Cuadernos de Coyuntura 32: Presente y Futuro de la Minería Nacional. Debate y Evaluación de un ciclo que Culmina* (CEDLA, 2021). The devastating impact of the wave of wildfires exacerbated existing tensions between different groups around competing, and at times incompatible, ways of managing land and local resources, as well as cultural practices and visions of future development. Particularly since the wildfire crisis in 2019, frictions have been underpinned by racist and discriminatory narratives, leading to growing social and ethnic stigmatisation.

Meanwhile, in 2019, for the first time the environmental crisis spilled into national political debates. The Bolivian presidential campaign was dominated by discussions on how to best manage the wildfire emergency. A major political crisis was triggered by mass protests against incumbent president Evo Morales, soon after the elections on the 20^th^ of October 2019, which culminated with his resignation and leaving the country. National political instability exacerbated tensions in the fire-affected regions, where different social and ethnic groups support competing political forces: highland migrants are strongholds of Morales’ party, ranchers and the agribusiness are the stronghold of the right-wing opposition, increasingly joined by lowland indigenous groups. Both the fire emergency and the political crisis have deepened and intensified local conflicts in the region, with numerous newspaper articles reporting inflammatory public discourse during rallies and mutual accusations and threats among different social groups. This context offered a unique opportunity to pilot FTR as a tool for transforming socio-environmental conflict.

The project relied on a close partnership and collaboration with a Bolivian organisation, Ciudadanía Bolivia (Ciudadanía), an NGO with extensive experience in applied research and community development. A team of three researchers and one logistics aid from Ciudadanía were integrated in the project team at an early stage. They led the logistics, organisation and coordination efforts for fieldwork preparation and implementation, and they were involved, at a later stage, in the production of research and art-based outputs (documentary film and photo exhibition) and their dissemination in Bolivia.

**Figure 1.**
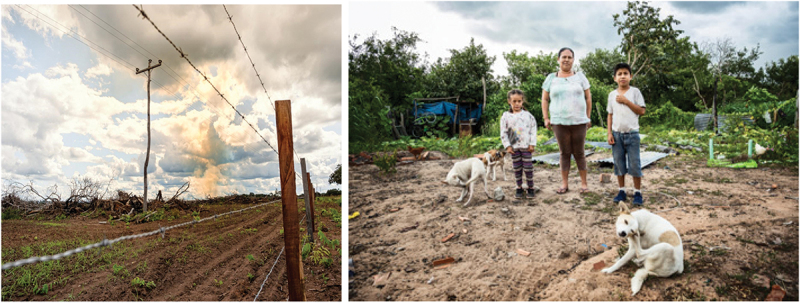
Vegetation is being burned to prepare the land for agriculture. A workshop participant and her two children, who were directly affected by the wildfire crisis.

Relying on Ciudadanía’s network in the Chiquitania region, preliminary contacts were established with community leaders and potential participants to present the project, the goals and participation conditions and requirements. This work led to the identification of 28 adults (15 men and 13 women) aged between 16 and 55 from 22 wildfire-affected communities (see Figure 1). The two essential criteria to participate were: a) living in Chiquitania and, b) having direct experience of wildfires. We strived to form a balanced group in terms of gender and age as well as to maximise the number of different social and ethnic groups represented. Ultimately, the group was formed by 20 indigenous people belonging to 14 different communities, 4 members of mixed indigenous/peasant communities, 2 members of migrant peasant settlements, and 2 urban dwellers.^36^^36^All participants were compensated for the time they spent working on the project. To resource the participation barriers often faced by parents of young children, particularly women, we offered the opportunity for children to accompany their parents and childcare was provided during workshop hours. Because of the widespread mutual distrust and stigmatisation, it was difficult to secure equal participation of all communities. For instance, the Mennonite groups we contacted declined to participate as these activities are not permitted for them.

Our FTR intervention lasted three weeks between March and April 2022. The research team deployed in Chiquitania was composed of a professor of international sciences, an applied theare consultant, a photojournalist and a filmmaker, all fluent in Spanish, and a team of two local researchers and one logistics and organisation aid from Ciudadanía. During a five-day workshop, the researchers shared the objectives of the action research and the group created four FT plays representing unresolved conflicts related to wildfires. At the end of the workshop, the research team, in consultation with the participants, selected two of these plays that were more closely linked to the research objectives and identified seven of the 28 participants to perform the plays during a two-week tour across wildfire-affected areas. Approximately 800 people attended 14 performances in 13 communities (three peasant migrants, five indigenous, three urban and two mixed). Fifty-five audience members intervened on stage and more than 450 offered alternatives to staged conflicts and assessed their feasibility in their specific social context.

For the purpose of this paper, we restrict our analysis to one of the two plays entitled *Pedro y Juanita* which was performed nine times in nine communities. We focus on this play because its plot directly touches on key issues under scrutiny: ethnic and social stigmatisation, the process of enemification of oppressed groups and the role of politically manipulated narratives in escalating violence. The data we analyse include the dramaturgy of the play and the audiences’ interactions (both embodied change strategies improvised on stage and after-action oral reflections) and the self-reflections of participants and the research team collected during the participatory evaluation sessions. We also draw from over 30 interviews with local experts and stakeholders (e.g. government officers, representatives of NGOs, local organisations and public authorities) on the causes, management and impact of wildfires in the region.

## The Forum Theatre for Reconciliation approach

The FTR approach is composed of the five steps we describe below, specifically highlighting the aspects that make this contextualisation of FT to conflict transformation different from Boal’s originating method.

### Building trust and active listening

**Figure 2.**
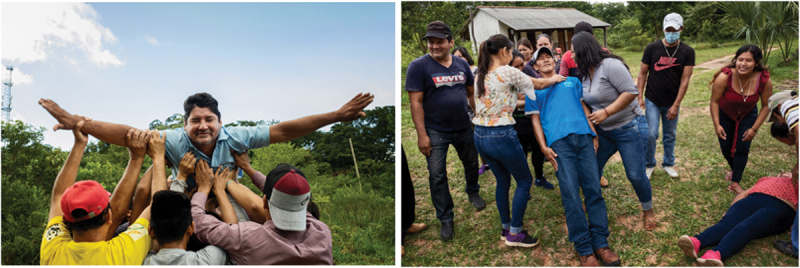
Trust and non-verbal listening games.

At the beginning of the workshop, the facilitator proposed theatrical exercises to build an interactive space of play, deep listening and non-judgement among the participants (Figure 2).^37^^37^Angelo Miramonti, *How to Use Forum Theatre for Community Dialogue. A Facilitator’s Handbook* (KDP, 2017). This step took approximately day one of the workshop (8 hours) and continued for approximately two hours each day for the following four days. Adapting Forum Theatre to conduct action research and foster peacebuilding has ethical implications. In this sense, we designed the process not only to break the oppression, but also to accompany all parties in recognising their wounds related to the conflict, honouring the feeling and rehumanising the other parties. Working on lived experiences of natural disasters also implied minimising the risk of revictimization and creating a safe space for sharing and processing traumatic memories. Building on the experience of working with Forum Theatre with victims of armed conflicts,^38^^38^Angelo Miramonti, ‘Healing and Transformation Through Arts: Theatre for Reconciliation’, *Educazione Aperta, Journal of Critical Pedagogy* 6 (2019): 40–60. the facilitator proposed theatrical exercises of trust, nonverbal listening and cooperation to build a symbolic container where these painful experiences could be shared.

While standard FT does not intentionally seek to build dialogue and empathy between the perceived oppressed and oppressors,^39^^39^Adrian Jackson, Interview with Julian Boal (Cardboard Citizens, 2020), https://www.youtube.com/watch?v=eRLqMsdCH2w&t=8s the first innovation of FTR is to intentionally gather individuals belonging to opposing parties who reciprocally perceive the Other as their antagonist, instead of homogenous groups of pre-identified ‘oppressed’ and their allies. To do so, FTR uses active listening and trust exercises with a mixed group as the first and essential step to reduce stereotypes and reciprocal enemification among the participants and allow them to empathically listen not only to the other parties’ ideas about the causes of the conflict, but to their lived experiences of unresolved oppression, in this case, related to wildfires. This moves away from standard FT games aimed at building solidarity and motivation to act among homogeneous groups of disempowered persons.

### Collective dramaturgy

In the second step, participants split into four subgroups and shared their lived experiences of wildfire-related oppressions (day 2 and 3). The aim was to foster intersubjective sharing of painful memories and the ‘re-authoring’ of the participants’ stories.^40^^40^Michael White and David Epston, *Narrative Means to Therapeutic Ends* (WW Norton & Company, 1990). The facilitator then invited the groups to choose one story (or to blend different stories) and enact them in a short play. While in FT this phase is based on choosing the themes perceived as most urgent by a homogenous group of oppressed, in FTR, this exercise of collective dramaturgy strives to identify an inclusive narrative around the conflict. This endeavour was a critical step in our workshop as a way of engaging in a positive interaction between indigenous and migrant participants who are often accusing each other of bearing responsibilities for wildfire disasters. While FT narrative and storylines typically represent a singular positioning or experience of oppression, FTR dramaturgy attempts to work across the situated narratives of opposing groups. The method tries not to approach conflict from a fixed position and avoids (as much as is possible) a preconceived idea of which character or group is oppressed. In FTR, we seek to explore the complexity of violence and how oppression can encompass different groups in different ways. The play *Pedro y Juanita* represents a telling example of this approach, which attempts to complicate the characters who are in conflict and blur an easy distinction between oppressors and oppressed. We can see this reframing in the dialogue and relationship between indigenous and migrant participants in the play *Pedro y Juanita*.

Pedro and Juanita are peasants living in the Bolivia’s highlands. The harvest is getting poorer and poorer, and Pedro proposes to his wife, Juanita, that they move to the Chiquitania in search for better land. Juanita is concerned about not having ownership rights for the land they will occupy, but Pedro pressures her and she accepts. Upon their arrival, a local officer of the National Institution for Agrarian Reform (INRA) explains that there is state-owned land available, but to obtain land rights, they would need to deforest and ‘make it productive’. In a few years, they would be able to claim settlement rights. Juanita has doubts, but Pedro convinces her to accept. The officer recommends that they do not burn before the first rain. Pedro and Juanita reach the assigned plot and start clearing trees. It is still the dry season, the rains are delayed, and they are keen not to burn before the first rain. But a manager of a neighbouring ranch approaches them and advises them to set fire straight away, when it is easier to burn, so they will be ready to plant their crops as soon as the first rain arrives. The rancher secretly plans to have Pedro and Juanita clear that land and eventually evict them so that he can claim the land for himself.

Pedro and Juanita decide to follow the advice because they need to generate income as soon as possible. But the fire gets out of control, spreading to the crops of a neighbouring indigenous community. Indigenous leaders travel to the government agency that grants burning permits (Forest and Land Inspection and Social Control Authority, ABT) to report the illegal settlement. An officer receives them and promises to carry out a site inspection the day after, but he does not comply. Indigenous leaders return to the office and ask to see the officer. He is there but he intimates a young employee to send them away. The employee tries to protest, but the manager threatens to fire her. Reluctantly, she acquiesces.

**Figure 3.**
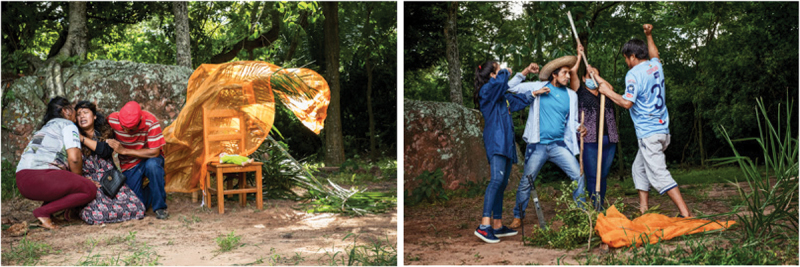
Rehearing the loss of a house in the wildfire. Rehearsing a violent confrontation between indigenous and peasant migrants.

Pedro and Juanita are left with only a few unburned plants to complete the clearing of their plot. Pedro feels the pressure to plant soon and goes ahead with the burning, despite Juanita’s reluctancy. On their way back from town, indigenous leaders see the smoke from the plot and confront the peasants claiming they are invading ancestral land. Pedro and Juanita argue that indigenous communities are just envious of their migrant entrepreneurship. A violent fight breaks out (Figure 3). Migrant and indigenous characters confront each other with machetes and hoes. The actors freeze.

### Exploring character complexity

The third step (day four of the workshop) in our adaptation of FT focuses on building characters’ biography, perspective and emotions. In standard FT, character building often centres around the oppressed and their potential allies, while the subjective motivations and complexities of the oppressors are not systematically and intentionally explored. The oppressor is often a character representing an entire hegemonic group^41^^41^Jackson, Interview with Julian Boal. and its construction is often limited to the identification of the prevailing oppressive strategies of this group and its ideologies of legitimation. Conversely, in our FTR pilot, we used a method called ‘Interview the Character’, a theatrical process inspired by Stanislavsky.^42^^42^Constantin Stanislavski, *An Actor Prepares* (New York: Routledge, 1989); and Miramonti, *How to Use Forum Theatre for Community Dialogue*. Each performer was invited to sit on a chair facing the other participants who interviewed the character asking questions about their childhood, their perceived identity, fears and worldviews. The facilitator invited the participants to give equal attention to each character and to explore their respective backgrounds and experiences with the same depth and complexity.

To generate empathy and mutual understanding, we also used a method we call ‘Swapping Roles’. While in FT the participants often belong to a homogeneous group, we purposedly invited workshop participants not to ‘play’ and embody their own lived experiences on stage but to swap roles with their perceived antagonists. Indigenous participants were invited to perform the experiences of peasant migrants and vice versa.

Both these methods are designed to support participants in the effort to feel the entangled and sometimes contradictory interiority of a character belonging to the opposing group, engaging in dialogue with members of that group through the theatrical fiction.

### Public performances

**Figure 4.**
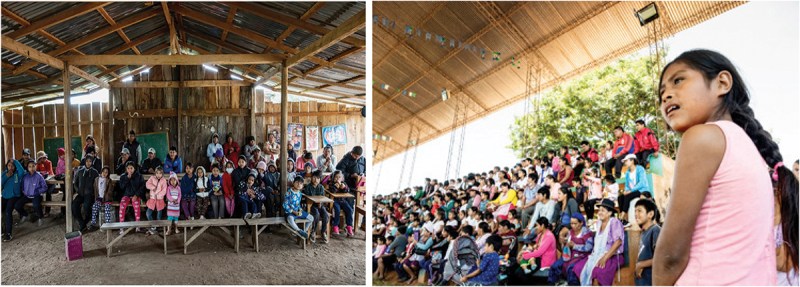
Audiences in rural and peri-urban areas.

In the fourth step, the shared dramaturgy and the characters created in the protected space of the workshop encountered the lived experiences of very diverse communities. *Pedro y Juanita* was performed in 9 communities (3 urban, 3 indigenous and 3 peasant migrants) where 30 spect-actors intervened on stage, replacing the characters they identified as oppressed and improvising strategies of transformation. More than 300 audience members discussed the feasibility of these staged interventions (Figure 4). This phase follows standard FT with one key difference: while in FT the facilitator invites the audience to replace the oppressed protagonist specifically, in FTR both the oppressed characters and the witnesses and potential allies (e.g. the employee) can be replaced: these are characters who, although not directly affected by the oppression, may have a realistic motivation to act in solidarity with the oppressed.

### Self-reflections

**Figure 5.**
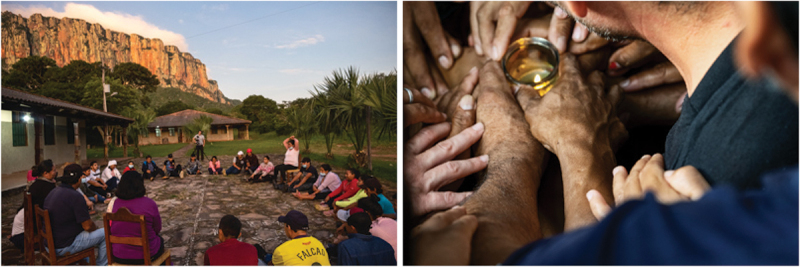
A moment of self-reflexion during the workshop. Ritual closure of the workshop.

During the workshop, 28 participants and the research team reflected on how drama prompted a re-encounter with their memories of wildfires and with the narratives of others. This step included brief oral evaluation sessions (of about 15 minutes) at the end of each day of the workshop, a 15-minute oral discussion after each public presentation, two meetings of one hour each during the tour and a closing ritual at the end of the three-week experience (Figure 5). Collecting and integrating feedback in the plot and in the characters’ personalities was also part of an ongoing dialogue throughout the rehearsal process. While in standard FT it is common for the group to reflect on the rehearsed strategies and their application in real life, in FTR, this exercise centred on the participants’ acknowledgement of responsibilities in co-creating the conflict and on the attitudes and behaviours that can contribute to processes of enemification and escalation of violence. Participants were invited to reflect on how enacting a shared narrative and embodying the enemy in front of diverse audiences had transformed them (even just a little) and motivated them to act differently. Reflecting on this experience, one indigenous participant, who embodied Pedro (a migrant peasant character), on stage said: ‘After performing several times my character, I understood that the migrants are as poor as we are, they are not the culprit of what is happening in Chiquitania’.

## A new drama-based approach to conflict transformation

In this section, we discuss the three main innovations of FTR and their theoretical and empirical implications. We argue these key features make this approach a distinctive drama-based peacebuilding method within the broader Theatre of the Oppressed methodology.

### Re-humanizing the other and the self through role swapping

One of the key innovations of FTR is the technique of Swapping Roles. The participants were invited to create and embody a character belonging to an opposing group. This process of ‘embodying the enemy’ invited the participants to mirror themselves in the dramatisation that the ‘other’ was doing of them and to realise that ‘they see us like that’. At the same time, this method invited the participants to become a mirror for others (‘I see you like this’). The participants’ comments on this experience demonstrate that embodying the complexities and contradictions of characters from an opposing community was instrumental in reframing the experience of the subject, inviting them to expand their capacity to *empathically imagine and feel* the perspective of the other. This implies both cognitively learning about the other’s subjective experience and emotionally feeling how the other experience their positioning in the conflict. This co-construction of characters based on shared lived experiences and role swapping was a dialogical process where, for example, an indigenous participant embodying a peasant had to listen to a peasant participant to learn how her character would feel, think and act in the situation on stage. Through role swapping, each participant was invited to teach the others about the ideology and desires of their own group and, at the same time, to listen to the others to learn how to embody their perspective in the character. This two-way dialogical process of character building was an important step for reframing the representations of the Self and the Other in the conflict. The character’s ‘mask’ allowed a protected space in which stereotyping, blaming and enemification could be expressed and incorporated by the performer, without necessarily identifying with them. This provided the participants with a symbolic mirror to critically assess the enemifying discourses of their own group, contributing to building the character, while also distancing themselves from such discourses.

Moving from embodying the enemy in the interview to performing them on stage was a further reinforcement of this mutual learning: it did not only imply cognitively understanding the perspective of the other but also emotionally feeling how their character (belonging to the opposing group) would react to the attitudes and behaviours of the performer’s own group. Having to respond to the unexpected strategies of spect-actors while remaining faithful to their character’s interior struggle reinforced the participants’ embodied understanding of the opposing group. The actor had to ‘embody the emotions and reactions of the enemy’ not only in the quiet setting of an interview but throughout a non-scripted dramatic action, where the ideology, fears and biography of the character were challenged to respond to unexpected alternatives coming from the audience. In this phase, the facilitator invited the actors not to rationalise how their character would act in that situation, but to ‘live the character’ and ‘feel’ the character’s reaction.

The embodiment of the character was an iterative process throughout the nine public performances. When some performers experienced uncertainties in their reactions, this prompted them to deepen their empathic connection with the character and to ask the advice of the participants from the opposing group. During the closing ritual, some participants shared that after embodying and performing a character belonging to their opposing community in front of external audiences, they felt the life of the character within themselves, and they would take this embodied character with them when returning to their communities. This aspect of the FTR approach fits in the emerging ‘embodied reconciliation’ agenda in peace studies and contributes to a paradigm shift towards an embodied approach to reconciliation. Through FTR tools (e.g. Role Swapping), the embodiment of the lived experiences and viewpoint of the enemy can foster intercommunal understanding and reconciliation.

During the *Pedro y Juanita’s* tour, audiences were invited to replace whichever character they perceived as oppressed in the situation. All nine audiences identified both Pedro and Juanita (migrants) and the two indigenous farmers as oppressed, together with the young employee as a possible allay. It is worth noting that in Scene One, Pedro was identified by most spectators as an oppressor of Juanita (because he puts pressure on her to migrate without adequate preparation), while he was identified as oppressed in Scene Two (where the rancher convinces him to burn before the first rain). Some characters (the ATB manager, the INRA officer, etc.) were not replaced because not recognised as oppressed, but their internal struggles and subjective motivations were explored during the Interviewing the Character. While in standard FT there is often an implicit assumption that empathy and solidarity should be channelled towards the oppressed and their urgent need for liberation, in FTR, the same complexity is acknowledged to each character, exploring their contradictions, irrespective of the role the dramaturgy attributes to them (which may not necessarily be the same role identified by audiences). This is a way to recognise the humanity of oppressors and the possibility for them to change behaviour, without condoning the dehumanising attitudes they are reproducing.

This approach attempts to avoid the enemification of the oppressor and their misrepresentation as ‘bad persons’. Their motivations and how the ideology of their group legitimate their behaviours were explored as any other. Adopting a maieutic dispositive, we focused on the cases when those with a consolidated hegemonic position are not ready to give up their oppressive role and fight to reproduce the oppressive *status quo*. We invited the audiences to explore the alternatives available to the disempowered characters (indigenous and peasants) and their privileged allies (the employee). We believe this strategy prevented (at least in part) the dehumanisation of the characters the audiences believe had an oppressive role. We do not assume that characters are incapable of undertaking a process of conscientization and behavioural change, but we choose to focus on the realistic scenario in which they will not ‘magically’ abandon their deeply engrained patterns of behaviour and ideologies of self-legitimation. The purpose of an FTR play is thus to explore ways to redistribute power between the characters as an initial step in the journey of reciprocal re-humanisation, assuming the abolition of the oppressor-oppressed roles as the long-term goal of this work.^43^^43^Freire, *Pedagogy of the Oppressed*. The fact that our intervention focused on environmental issues also allowed to build common ground around exogenous drivers to all local groups, such as changes in climate patterns, which were acknowledged by many of the participants from different groups.

### Complexifying local narratives: creating discursive inclusion on lived and structural causes

During the workshop, participants shared their lived experiences of conflicts related to wildfires through embodied narratives. It was relatively easy to identify shared stories of the devastating consequences of wildfires, but much harder to find a common narrative about the trigger and responsibilities of this socio-environmental crisis. Through theatrical exercises, it became apparent that differences were linked to the diverging views of each group on the causes of their oppression. We were confronted with the challenge of valuing people’s diverging viewpoints and validating their testimonies while intertwining them in a complexified and non-polarising plot. This dialogical search for a common narrative around the conflict was one of the key results of the workshop. Listening to the lived experiences of the other group was the beginning of an ‘improbable dialogue’ and a step towards the co-creation of a ‘third narrative’ around wildfires. Co-creating with ‘the enemy’ led to an inclusive narrative of the conflict in which every participant could identify. *Pedro y Juanita* is the result of this encounter.

Crucially, in this process of narrative convergence and mixing, it was possible to address some of the biases that we noticed in early versions of the story. Two emerged and became relevant throughout our intervention. Firstly, we noted that the dramaturgy was initially biased by the indigenous viewpoint (most participants) whereby indigenous communities were the oppressed and migrant peasants the invaders: ‘We are indigenous at the service of the forest, we don’t destroy, we protect the forest. Peasants are those who destroy widely, mechanisation, enterprises, Mennonites and people with money’ Migrant participants instead considered they were legitimately occupying state-owned land, while indigenous communities were envious of their entrepreneurial skills, hard work and economic achievements: ‘Peasant and indigenous people have different ways of thinking and living. These is where conflict arises. They [indigenous] work with big landowners and they are happy with the little they gain. We [peasant] resist being manipulated. We fight. We strive to have a bit more every day. We work hard for that, and this generates the envy [of indigenous people]’.

Secondly, the role of other stakeholders and of broader causes of wildfires were almost completely absent from local participants’ narratives. Most of them initially identified the undisciplined migration of peasants from the highlands and their irresponsible burning practices as the main cause of fires. And yet, we know from research (including our own fieldwork) that wildfires in Bolivia are complex phenomena, linked to at least four structural factors: climate change (particularly long and intense dry seasons and el Niño cycles); expansion of the agrarian frontier in response to a growing demand for agricultural and livestock products; new government policy which incentives internal migration towards forested lowlands; and weak fire management and prevention strategies, coupled with institutions plagued with corruption, underfunding and inefficiencies.^44^^44^Fundación Tierra, 2019; Tahia Devisscher, Yadvinder Malhi and Emily Boyd, ‘Deliberation for Wildfire Risk Management: Addressing conflicting views in the Chiquitania, Bolivia’, *The Geographical Journal* 185, no. 1 (2019): 38–54; and Minerva Singh, Shivam Sood, and Matilda Collins, ‘Fire Dynamics of the Bolivian Amazon’, *Land* 11, no. 9 (2022): 1436.

These considerations raised the issue of what to do when the FTR facilitator(s) feel that some systemic causes are absent in this kind of theatrical work. This is particularly evident when dealing with socio-environmental issues, where cause-effect nexuses are complex and often intertwined with broader changes (linked to for example climate and precipitation patters or natural resource global markets and governance). We questioned how much external information can or should facilitators and researchers add to the lived experiences of conflict. How can external agents contribute to broadening local understanding of what causes wildfires without manipulating and diminishing local ownership of the play? How can we find complementarity between the expertise that comes from having personally experienced wildfires – sometimes with extremely painful consequences – and other forms of expertise coming from scientific evidence and research? There are no easy answers to such questions, and these concerns accompanied us throughout the process of creation and performance of *Pedro and Juanita*. Our preliminary answer was inspired in Freire’s maieutic dialogue: the facilitator invited the participants to share their lived experiences and the members of the research team to share research findings on wildfires, avoiding a hierarchical and one-sided transfer of knowledge and valuing the knowledge of both sides as equally important.

In the effort of including a wide range of perspectives, the dramaturgy of *Pedro y Juanita* was not defined once and for all. It was iterative and open. In some occasions, audience dialogue provided critical insight and material which was then folded into a revised plot, one perhaps closer to the experience of different groups. For example, during the performance in a peasant community, the audience confronted the actors and the facilitator, claiming that the play was unfairly blaming peasants for provoking wildfires. This prompted a new reflection and modification of the plot: it was made clearer that Pedro and Juanita had to migrate because of reduced fertility in the highlands and that, upon arrival, they had limited savings and urgently needed to plant crops to survive. The rains are delayed due to changes in climate patterns, and a rancher (representing powerful economic actors) is enacting strategies of land dispossession of vulnerable communities in response to changing economic interests linked to demand in global markets. As more structural causes of fire were not dominant in participants’ stories initially, they gained importance in later stages. Adding depth and complexity to the scene was an iterative process throughout its public performances.

It is worth noting that constructing a complex narrative not only challenged the understanding of local participants and audiences but also those of the research team. This method proved particularly relevant to complement other qualitative methods of inquiry^45^^45^Rosaline Barbour, *Introducing Qualitative Research. A Student’s Guide to the Craft of Doing Qualitative Research* (Sage Publications, 2007); and Bob Matthews and Liz Ross, *Research Methods: A Practical Guide for the Social Sciences* (Pearson Education Ltd, 2010). with drama-based methodologies.^46^^46^Mandy Archibald, ‘Interweaving Arts-Based, Qualitative and Mixed Methods Research: Showcasing Integration and Knowledge Translation Through Material and Narrative Reflection’, *International Review of Qualitative Research* 15, no. 2 (2022): 168–98; Luisa Enria, ‘Co-producing Knowledge through Participatory Theatre: Reflections on Ethnography, Empathy and Power’, *Qualitative Research* 16, no. 3 (2016): 319–29; and Randee Lawrence, ‘Dancing with the Data: Arts-Based Qualitative Research’, in *Handbook of Research on Scholarly Publishing and Research Methods*, ed. V. Wang (IGI Global, 2015), 141–54. In situations of high social tensions, participatory drama-based approaches can effectively contribute to generating information, while enabling conflicts to be explored in a safe environment. Theatrical games and activities, while not directly part of the data-gathering process, proved crucial to build a shared space of mutual trust and respect where it was easier for participants to share difficult stories and life experiences.^47^^47^Johnston and Pratt, *Migration in Performance*. In contexts where high levels of distrust and tension prevail, this method can go much further than traditional qualitative methods such as interviews and focus groups. These methods are not mutually exclusive and, as we have illustrated, can complement the efforts of gathering an accurate picture of local complexity.

Moving from cognitive to emotional aspects, this complexification of narratives showed that theatre can be an especially effective space in which to tell stories of loss and suffering, entering emotionally difficult encounters with the narratives of others on stage. This encounter was made possible by the liminal and symbolic nature of the stage, where narratives of oppression created across the divides were enacted, actors coming from opposing communities embodied the reasons of their enemies and the audience was invited to ‘write another ending’ with their bodies, words and actions, confronting the resistance of oppressive characters in a safe space of critical reflection and conscientization.

### Devising collective action across divides

The combination of reciprocal re-humanisation with the complexification of narratives opens the space for ‘improbable dialogues’^48^^48^John Paul Lederach, *The Moral Imagination: The Art and Soul of Building Peace* (Oxford: Oxford University Press, 2005). and for building ‘improbable alliances’ to respond to the common challenge of wildfires. The strategies enacted by audience members during the performances demonstrate a widespread awareness for urgent collective responses to wildfires.

While some spect-actors blamed the migrants for invading their land and proposed to violently stop them: ‘We are being too accommodating with people from the highlands’; ‘We have to unite and stop the subjugation of our land’; others acknowledged that ‘[migrants] are not to blame for what happens, they come here because they are poor’ and that ‘migrants have an urgency to produce and burn without permit out of necessity’. Some acknowledged that migrants ‘cannot be prevented from arriving, but we can integrate them in our communities’. As part of the strategies to integrate newcomers, some spect-actors proposed involving them in wildfire prevention and response: ‘Indigenous authorities should meet the newly arrived migrants and invite them to collaborate on issues of common interest, like fire preparedness’ and ‘We have to teach the migrants how to burn’.

Moving from situational short-term responses to more institutional approaches, some spect-actors proposed that their elected community leaders should engage with public authorities to enforce the existing laws on burning permits. Others questioned the role of INRA in providing informal deforestation permits on state-owned land and the attribution of property rights to informal settlers. It was proposed that communities collectively urge authorities to enforce stricter burning practices and control: ‘The INRA should inform the migrants about the burning rules and asks them to meet with the neighbouring communities [before burning]’. Finally, some spect-actors expressed their discomfort with some proposed strategies because they were highly situational and failed to address the economic interests invisibilized by politically motivated narratives: ‘The problem is the big capitals coming to the region for agriculture and [to exploit] biodiversity, not the poor coming from the highlands. The poor are all being affected: they have to unite’; ‘The burning [of the dry forest] is being authorised to give land to large investors’. While structural long-term causes of wildfires, such as climate change and global markets, were the least mentioned among the workshop participants, audiences, especially in urban areas, showed a greater awareness and engagement with broader processes. This should encourage a reflection on the divide that often exists in dissemination of knowledge to more marginalised rural communities, and to the difficulties for these communities to make their voices and reasons heard more broadly to local authorities, fellow citizens, and scholars alike.

## Conclusions

We presented Forum Theatre for Reconciliation as a new drama-based action research approach, specifically designed for researchers and artists working in situations of conflict. FTR offers new tools to work with these groups with the aim of facilitating empathic reciprocal understanding so that shared collective strategies might be identified.

Juggling the conflicting agendas of peacebuilding and knowledge co-production can generate tensions and synergies between action and research that are not always smooth and easy to achieve.^49^^49^Susan H. Allen and Victor Friedman, ‘An Emerging Conversation Between Action research and Conflict Transformation’, *Action Research* 19, no. 1 (2021): 3–8; and Miren Larrea, ‘We are not Third Parties: Exploring Conflict between Action Researchers and Stakeholders as the Engine of Transformation’, *Action Research* 19, no. 1 (2019): 110–25. While both the research team and the local partner were generally perceived as impartial outsiders and not favouring any of the conflicting parties, during fieldwork and creative process, we had to take the role of mediators of tensions that arose between the indigenous majority and the migrant minority. We also had to confront uneasy situations where the play we presented triggered protests from the audience. This highlighted the fluidity of our positionality as scholars and peacebuilding practitioners and posed some challenges in maintaining this double role.^50^^50^Alexander Cromwell and Margarita Tadevosyan, ‘Deconstructing Positionality in Conflict Resolution: Reflections from First-Person Action Research in Pakistan and the South Caucasus’, *Action Research* 19, no. 1 (2021): 37–55. In this sense, it would be important for researchers and facilitators engaging in FTR to undertake training in mediation and conflict management.

This experimentation of FTR also carries some limitations and further challenges. Our experience working on socio-environmental conflicts allowed to gain access to often traumatic lived experiences of wildfire disasters as the primary source material for theatrical creation, with the advantage of creating stories which directly talked to audiences affected by wildfires. Yet these dialogues mostly focused on highly situational and short-term responses, reflecting the sense of urgency perceived by local actors, while more systemic causes and long-term structural interventions remained broadly unexplored. Legislative Theatre^51^^51^Augusto Boal, *Legislative Theatre. Using Performance to Make Politics* (London: Routledge, 1998). can offer a valid alternative or complementary approach to explore structural and long-term root causes of wildfire crises and to design policy-driven responses. Using Legislative Theatre to engage communities in self-legislation would also increase the sustainability of the institutional and behavioural changes reharled during the performances.

A second limitation of FTR is the fact that this kind of intervention is often constrained by strict schedules and short-term fieldwork where it is hard to grasp deeper and broader political, economic and ecological processes. While it is always recommended to integrate empirical findings with existing academic literature, sometimes an in-depth analysis of (social) media might shed light on mainstream collective narratives. In our case, for instance, it was clear that participants were deeply influenced by (often politically motivated) mainstream narratives, which circulated extensively through social and traditional media. For this purpose, we recommend experimenting with other drama-based methodologies for deconstructing narratives using other Theatre of the Oppressed techniques like Newspaper Theatre to unpack media manipulation.

A third challenge was the limited time to explore characters’ ideology and worldview. While swapping roles and interviewing the character proved effective, we also recommend applying Rainbow of Desire^52^^52^Augusto Boal, *Rainbow of Desire: The Boal Method of Theatre and Therapy* (London: Routledge, 1994). for an in-depth exploration of incarnated representations of the self and the other.

A more comprehensive methodological paradigm to contextualise Theatre of the Oppressed to conflict transformation with mixed groups of conflicting parties could include:

1. Listening and trust exercises, to open a space of dialogue between the parties;
2. Newspaper Theatre, to deconstruct media-driven narratives and highlight structural causes of wildfires;
3. Rainbow of Desire, to explore internalised conflicts of the participants and characters;
4. Forum Theatre for Reconciliation, to identify situational and grassroot responses to wildfires;
5. Legislative Theatre, to institutionalise wildfire preparedness responses.

We have discussed the innovative potential of FTR through the analysis of its implementation in the case of wildfire-related conflicts in Bolivia. We identify four key lessons based on this experience.

First, conflict transformation requires addressing mutually dehumanising narratives and embodying different worldviews, fears and aspirations. Working theatrically with people who have divergent experiences of conflict open possibility for sharing experience and understanding in transformative ways. Secondly, deconstructing politically motivated discourses requires co-creating an inclusive narrative addressing both the root causes and immediate consequences of conflict, combining the lived experiences of people affected by wildfires with scientific evidence in a maieutic and non-hierarchical dialogue. Building a new narrative of the self, the other and the conflict should not be a ‘banking’ transfer of information^53^^53^See note 43 above. – colonising the other with the well-intended narratives of a self-appointed élite – but a dialogical and maieutic co-construction of meaning to inform action. Third, rehumanising and building inclusive narratives prepares audiences for collective action across perceived ideological divides. Fourth, FTR demonstrates great potential as a method for social research on conflict-related issues. Theatre is a means of doing research, and the dramaturgical process and public performances offer unique insight into collective and individual experiences of crisis.

With intensifying extreme climatic events characterised by multiple economic, social and political vulnerabilities, FTR offers a valuable tool. While new technologies have dramatically enhanced our knowledge and understanding of wildfires from above (mainly through satellite images), it is equally important to keep eyes and feet on the ground, among the many communities and peoples whose lives have been dramatically impacted by extreme fires. Through their lived experiences, we can gain a better understanding of these changes. FTR and Theatre of the Oppressed more generally offer a powerful way to approach these realities by acknowledging the importance of individual and collective experiences. Using words, but mostly embodied images and stories, we can grasp the complexity and give credit to the multiple perspectives, while at the same time experimenting with the underappreciated role of artistic imagination in building peace.

## Photos

All photos were taken by Max Hirzel, see https://maxhirzel.photoshelter.com/index.

